# Predicting membranous nephropathy remission: a nomogram based on early dynamic biomarkers

**DOI:** 10.3389/fmed.2026.1783016

**Published:** 2026-07-20

**Authors:** Chendan Wang, Cheng Hu, Miao Zhang, Rongshan Li

**Affiliations:** 1 The Second Clinical Medical College of Shanxi Medical University, Taiyuan, Shanxi, China; 2 The Fifth Clinical Medical College of Shanxi Medical University, Taiyuan, Shanxi, China; 3 Sanmenxia Central Hospital, Sanmenxia, Henan, China

**Keywords:** anti-PLA2R antibody, dynamic monitoring, membranous nephropathy, nomogram model, prognosis prediction, proteinuria

## Abstract

**Background:**

Traditional prognostic assessment of membranous nephropathy (MN) relies on baseline static indicators, which do not allow for the dynamic monitoring of early treatment response. This study aims to evaluate whether early changes in anti-PLA2R antibody titers, 24-h urine protein quantification, and serum albumin levels can predict 12-month remission and to develop a nomogram model to facilitate clinical decision-making.

**Methods:**

A retrospective analysis was conducted on 144 patients with PLA2R-related MN treated with rituximab between 2022 and 2025. Patients were categorized into remission (*n* = 86) and non-remission (*n* = 58) groups based on 12-month outcomes. Dynamic changes in biomarkers were analyzed at 1 and 6 months post-treatment. Risk factors were identified using Lasso-Logistic regression, and a nomogram was constructed and evaluated using receiver operating characteristic (ROC) curves and calibration plots.

**Results:**

At 1 month post-treatment, non-remission patients demonstrated a significantly lower percentage decline in anti-PLA2R antibodies (47.26% vs. 85.74%, *p* < 0.001), less reduction in 24-h urinary protein (4.5% vs. 42.5%, *p* < 0.001), and a smaller increase in serum albumin (6.47% vs. 17.06%, *p* < 0.001). Multivariate analysis identified 1-month urinary protein (OR = 1.374), anti-PLA2R decline (OR = 0.944), and albumin increase (OR = 0.967) as independent predictors of remission. The 1-month nomogram showed excellent discrimination (AUC = 0.905) and strong calibration, with predictive value comparable to 6-month monitoring.

**Conclusion:**

Dynamic changes in anti-PLA2R antibodies, urinary protein, and serum albumin at 1 month are effective predictors of 12-month remission in MN. This nomogram model enables early risk stratification and the optimization of personalized treatment strategies.

## 1 Introduction

Membranous nephropathy (MN) is one of the most common causes of nephrotic syndrome in adults. It is characterized by immune complex deposition in the glomerular basement membrane and podocyte damage, leading to massive proteinuria and hypoalbuminemia (1). In 70–80% of cases, the exact cause remains unknown, and this form of the disease is referred to as primary membranous nephropathy (PMN). In recent years, the incidence of MN has increased, partly due to environmental pollution and the aging population (2). Some patients progress rapidly to end-stage renal disease (ESRD) within 5–10 years (3). Therefore, early identification of patients with a poor prognosis and timely initiation of immunosuppressive therapy are of significant clinical importance.

The M-type phospholipase A2 receptor (PLA2R) is the primary target antigen in PMN, and anti-PLA2R antibodies can be detected in the serum of approximately 70–80% of PMN patients (4). These antibodies have been confirmed to be specific for disease diagnosis, as well as indicators of disease activity and prognosis (2, 5, 6). In 2021, the KDIGO guidelines recommended using anti-PLA2R antibody levels as a biomarker for assessing treatment response in MN, with evaluations to be conducted at 3 and 6 months (7). The 2023 KDOQI US Commentary on the KDIGO guidelines further emphasized that monitoring anti-PLA2R antibody levels should focus on trends rather than absolute thresholds (8). Current research on MN prognosis generally relies on baseline static indicators (such as baseline PLA2R antibody titer, proteinuria levels, and estimated glomerular filtration rate (eGFR)), which are limited in their ability to dynamically reflect changes in disease activity during treatment. Therefore, elucidating the correlation between changes in anti-PLA2R antibody levels and MN prognosis has practical significance for optimizing clinical decision-making. To address this gap, this study retrospectively analyzed 144 PLA2R-related MN patients treated with rituximab (RTX) at Shanxi Provincial People’s Hospital, systematically evaluating the predictive efficacy of dynamic indicators before and in the early stages of treatment for non-remission at 12 months. A nomogram model was constructed to provide a scientific basis for the early identification of high-risk patients and adjustment of treatment strategies in clinical practice.

## 2 Research subjects and methods

### 2.1 Research subjects

This study is a retrospective cohort study. It included patients with MN who underwent regular follow-up at Shanxi Provincial People’s Hospital from January 2022 to January 2025.

Inclusion criteria: (1) Age ≥ 18 years; (2) diagnosis of PLA2R-related primary membranous nephropathy (PMN) based on either (a) kidney biopsy demonstrating typical histological features, or (b) positive serum anti-PLA2R antibodies with nephrotic syndrome in patients who did not undergo biopsy. (3) No prior use of glucocorticoids, immunosuppressants, or biologic agents before diagnosis; (4) At least 12 months of follow-up at our hospital.

Exclusion criteria: (1) Secondary membranous nephropathy; (2) Patients who had received glucocorticoids, immunosuppressants, or biologic agents before diagnosis; (3) Patients who had undergone kidney transplantation; (4) Patients who did not complete regular clinical follow-up.

### 2.2 Research methods and data collection methods

Collect general and clinical data of included patients, including: gender, age, height, weight, concurrent hypertension, diabetes, treatment regimen, and body mass index (BMI); laboratory indicators including PLA2R antibody levels, 24-h urine protein quantification, serum albumin, hemoglobin, white blood cell count, neutrophil count, platelet count, lymphocyte count, total cholesterol, triglycerides, blood urea, serum creatinine, eGFR, and D-dimer levels before treatment, 1 month after treatment. Use of RAASi, diuretics, statins, and anticoagulation therapy was also documented at each time point. Serum anti-PLA2R antibody levels were measured using a commercially available enzyme-linked immunosorbent assay (ELISA) kit. The assay was performed according to the manufacturer’s instructions. Antibody levels were expressed in RU/mL. A value of ≥14 RU/mL was defined as positive, levels below 14 RU/mL were considered negative. All measurements were performed by technicians blinded to patient clinical outcomes.

### 2.3 Treatment protocol

All patients received rituximab (RTX) as initial immunotherapy. The RTX regimen consisted of four weekly infusions of 375 mg/m^2^. Premedication with intravenous methylprednisolone was administered prior to each RTX infusion to prevent infusion-related reactions. Routine B-cell monitoring was performed during follow-up. Retreatment with RTX was considered at 6 months only for patients who had not achieved complete remission (CR) or partial remission (PR) according to the efficacy criteria defined in section 2.4.

All patients received standardized supportive care throughout the follow-up period. Renin-angiotensin-aldosterone system inhibitors (RAASi), including angiotensin-converting enzyme inhibitors (ACEIs) or angiotensin receptor blockers (ARBs), were administered at maximally tolerated doses to achieve a target blood pressure of <130/80 mmHg, unless contraindicated. Diuretics (primarily loop diuretics) were used as needed to manage edema and volume overload. Statins (mainly atorvastatin or rosuvastatin) were prescribed for patients with low-density lipoprotein cholesterol >2.6 mmol/L. Anticoagulation therapy (low-molecular-weight heparin or warfarin) was considered for patients with serum albumin <25 g/L and additional risk factors for thromboembolism (e.g., proteinuria >8 g/24 h, or prior thromboembolic events). The use and dosage of all supportive medications were documented at baseline and at each follow-up visit.

### 2.4 Efficacy and grouping criteria

Complete remission (CR) was defined as 24-h urine protein quantification < 0.3 g/24 h, serum albumin ≥ 40 g/L, and normal serum creatinine levels. Partial remission (PR) was defined as 24-h urine protein quantification between 0.3 g and 3.5 g/24 h, with at least a 50% reduction from baseline, improvement or normalization of serum albumin, and stable serum creatinine levels. Non-remission (NR) was defined as 24-h urine protein quantification ≥ 3.5 g/24 h, or a < 50% decrease from baseline in urine protein, or a ≥ 25% decrease in eGFR.

### 2.5 Definition of the exposure of interest


$$\begin{array}{l} \text{Change value of antibody}=\text{Antibody titer before treatment}\\ -\text{Antibody titer}\;1\;\text{month after treatment} \end{array}$$

$$\begin{array}{l} \text{Change value of urinary protein quantification}=\\ 24-h\;\text{urinary protein quantification before}\;\\ \text{treatment}-24-h\;\text{urinary protein}\;\\ \text{quantification}\;1\;\text{month after treatment} \end{array}$$

$$\begin{array}{l} \text{Change value of albumin}=\text{Albumin}\;1\;\text{month after treatment}\\ -\text{Albumin before treatment} \end{array}$$

$$\begin{array}{l} \text{Relative percentage}\;\\ \text{of antibody decrease}={\left(\begin{array}{l} \text{Change value of antibody}/\\ \text{Antibody titer before treatment} \end{array}\right)}^{*}\;100\% \end{array}$$

$$\begin{array}{l} \text{Relative percentage of}\;\\ \text{urinary protein decrease}={\left(\begin{array}{l} \text{Change value of urinary}\;\\ \text{protein quantification}/\\ 24-h\;\text{urinary protein}\;\\ \text{quantification before}\;\\ \text{treatment} \end{array}\right)}^{*}\;100\% \end{array}$$

$$\begin{array}{l} \text{Relative percentage}\;\\ \text{of albumin increase}={\left(\begin{array}{l} \text{Change value of albumin}/\\ \text{Albumin before treatment} \end{array}\right)}^{*}\;100\% \end{array}$$

### 2.6 Statistical methods

Data analysis was conducted using SPSS 27.0 software. For measurement data that followed a normal distribution, results were expressed as mean ± standard deviation (x̄ ± s), and comparisons between the two groups were made using independent samples *t*-tests. For measurement data that did not follow a normal distribution, results were expressed as median (interquartile range, IQR: Q1, Q3), and comparisons between the two groups were made using the Wilcoxon rank-sum test. Count data were expressed as frequency or percentage, and comparisons between the two groups were made using the *χ*^2^ test. Lasso-Logistic regression analysis was used to identify factors associated with non-remission in MN patients after 12 months. Based on the results of the Logistic regression analysis, a nomogram model for predicting non-remission in MN patients after 12 months was constructed using R software (version 4.4.3). The performance of the model was evaluated using the receiver operating characteristic curve (ROC curve) and the Hosmer–Lemeshow goodness-of-fit test, while the clinical utility was assessed using calibration curves. A two-sided *p*-value of less than 0.05 was considered statistically significant.

With 144 patients included in the study and 58 non-remission events, the sample size was determined by the availability of eligible patients during the study period rather than by a prospective power calculation. For logistic regression model development, a commonly recommended minimum of 10 events per variable (EPV) was considered. Our final multivariate model included 3 independent predictors (1-month urine protein, percentage decrease in anti-PLA2R antibodies, and percentage increase in albumin), yielding an EPV of approximately 19.3, which exceeds the conventional threshold of 10, suggesting adequate sample size for reliable model estimation. Furthermore, LASSO regression was employed for predictor selection to reduce the risk of overfitting by shrinking coefficients and performing automatic variable selection.

Due to the retrospective nature of this study, missing data were present for some laboratory indicators, particularly at the 3-month follow-up time point (approximately 85% of patients had incomplete data at 3 months). Given this high proportion, the 3-month time point was excluded from the analysis. For the 1-month and 6-month time points, missing data were minimal (<5%). Complete case analysis was performed, meaning that patients with missing data for a given time point were excluded from analyses involving that time point. No imputation methods were applied. To assess potential bias, we compared baseline characteristics between patients with and without complete follow-up data; no significant differences were observed, suggesting that missingness was unlikely to introduce substantial bias.

The predictive performance of the 1-month and 6-month models was compared using DeLong’s test for paired ROC curves. A two-sided *p*-value of less than 0.05 was considered statistically significant.

## 3 Results

### 3.1 Comparison of clinical data between the remission and non-remission groups at baseline

This study included a total of 144 patients with MN, of whom 86 were classified into the remission group and 58 into the non-remission group. Analysis of the baseline characteristics of the two groups revealed statistically significant differences in age and hypertension prevalence. Specifically, patients in the non-remission group were older and had a higher incidence of hypertension. No statistically significant differences were observed between the two groups in terms of gender, BMI, or diabetes history (Supplementary Table 1).

### 3.2 Laboratory indicators at 1 month after treatment

One month after treatment, statistically significant differences were observed between the two groups in terms of antibody levels, antibody conversion to negative, 24-h urine protein quantification, serum albumin levels, relative percentage decrease in antibodies, relative percentage decrease in urine protein, relative percentage increase in albumin, changes in albumin levels, and changes in 24-h urine protein quantification. Specifically, the non-remission group exhibited higher antibody levels, higher 24-h urine protein quantification, lower serum albumin levels, smaller relative percentage decreases in antibodies and urine protein, smaller relative percentage increases in albumin, as well as smaller changes in albumin levels and urine protein quantification. There was no statistically significant difference between the two groups in terms of changes in antibody levels (Supplementary Table 2).

### 3.3 Laboratory indicators at 6 months after treatment

Six months after treatment, statistically significant differences were observed between the two groups in terms of antibody conversion, 24-h urine protein quantification, serum albumin levels, and eGFR. Specifically, in the non-remission group, the proportion of patients with persistent positive antibodies was higher, 24-h urine protein quantification was higher, serum albumin levels were lower, and eGFR values were lower (Supplementary Table 3).

### 3.4 Filtering independent influencing factors before treatment

Binary logistic regression analysis of the baseline general data and laboratory indicators of patients before treatment identified antibody titer level (OR: 2.635, 95% CI: 1.162--5.798, *p* = 0.020), eGFR (OR: 0.981, 95% CI: 0.965--0.998, *p* = 0.025), cholesterol level (OR: 1.135, 95% CI: 1.034--1.246, *p* = 0.008), and concurrent hypertension (OR: 2.606, 95% CI: 1.073--6.328, *p* = 0.034) as independent predictors of poor prognosis (Supplementary Table 4).

### 3.5 Independent influencing factors after one month of treatment

**Figure 1:**
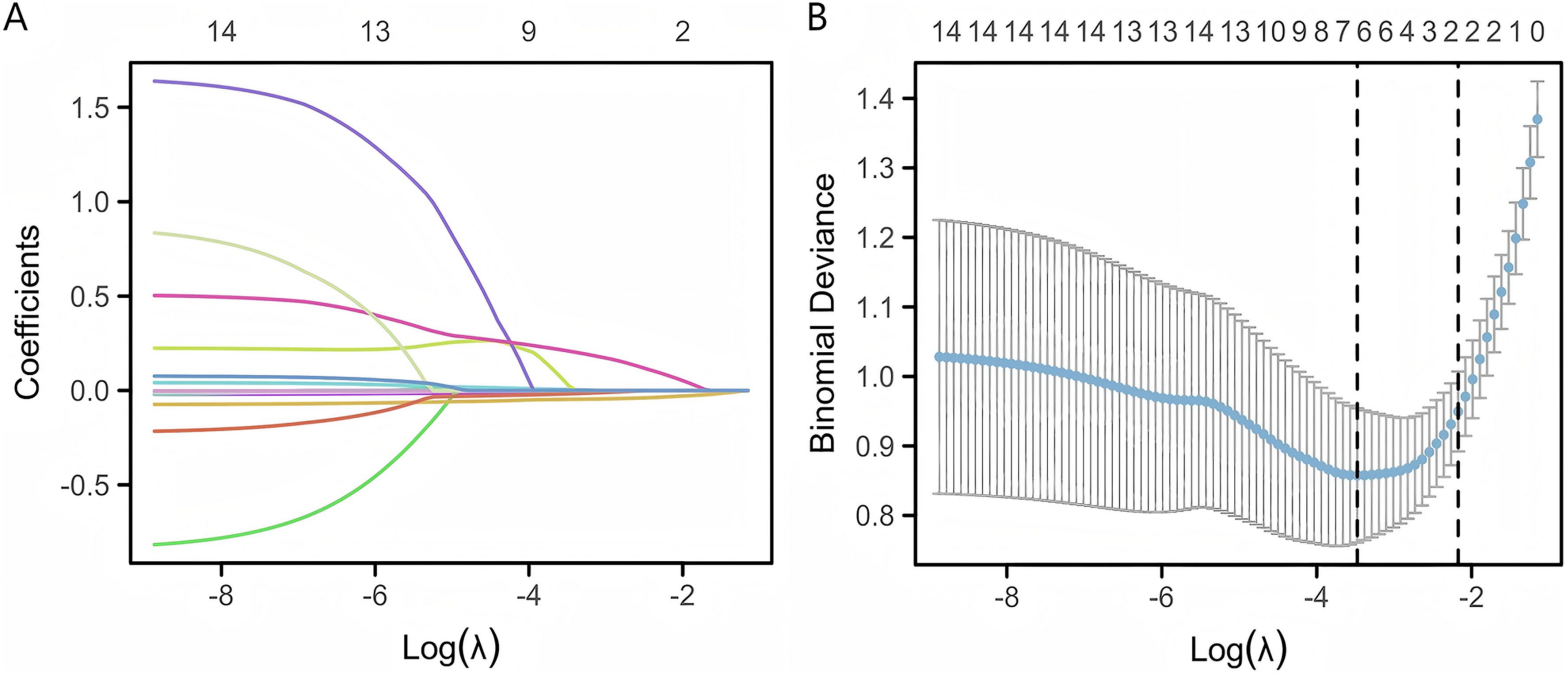
Screening independent factors associated with 12-month non-remission in MN patients using LASSO regression analysis. **(A)** Regularization path of LASSO regression coefficients. The *x*-axis represents the log-transformed regularization parameter *λ*. Each colored line represents the coefficient trajectory of a clinical variable. **(B)** Ten-fold cross-validation curve for LASSO regression. The *x*-axis shows log(*λ*), and the *y*-axis shows binomial deviance. The vertical dashed line indicates the optimal *λ* value selected based on minimum mean squared error.

**Figure 2:**
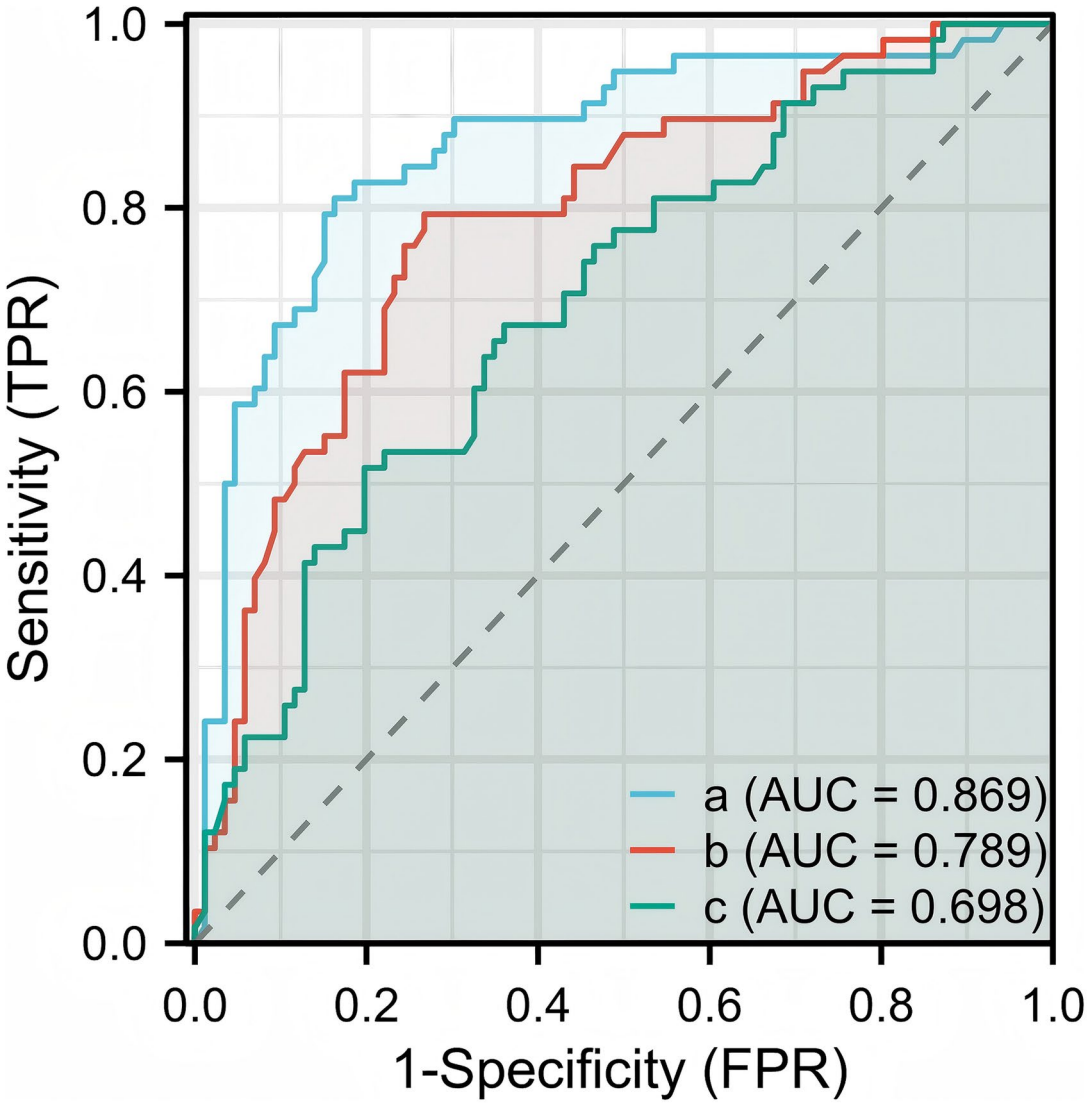
Predictive power of independent factors at 1 month after treatment for non-remission at 12 months. The ROC curves show the predictive performance of the three independent factors identified by multivariate logistic regression: percentage decrease in anti-PLA2R antibodies (blue line, AUC = 0.869), 24-h urine protein quantification at 1 month (red line, AUC = 0.789), and percentage increase in albumin (green line, AUC = 0.698). The diagonal gray dashed line represents the reference line (AUC = 0.5). Among the three factors, the percentage decrease in anti-PLA2R antibodies demonstrated the strongest predictive power.

To optimize model fitting performance, the study determined the regularization parameter *λ* corresponding to the minimum error value through cross-validation methods under the mean squared error evaluation metric (Figure 1). LASSO regression analysis identified a total of 7 variables: history of hypertension, age, eGFR, relative percentage decrease in antibodies, 24-h urine protein quantification at 1 month, relative percentage decrease in urine protein, and relative percentage increase in albumin. These variables were included in multivariate logistic regression analysis, which revealed that 24-h urine protein quantification at 1 month after treatment (OR = 1.374, 95% CI: 1.158–1.630, *p* < 0.001), percentage decrease in antibodies (OR = 0.944, 95% CI: 0.922–0.967, *p* < 0.001), and percentage increase in albumin (OR = 0.967, 95% CI: 0.937–0.998, *p* = 0.035) were independent predictors of non-remission after 12 months (Table 1). Among these three independent factors, the percentage decrease in antibodies significantly outperformed the other two in terms of predictive power (Figure 2).

**Table 1:** Multivariate logistic regression analysis of 1-month post-treatment outcomes predicting non-remission at 12 months in patients with MN.

Variable	*β*	OR (95% CI)	*p*-value
Hypertension			
Yes		Reference	
No	−0.618	0.539 (0.166–1.750)	0.304
Age	0.017	1.017 (0.967–1.070)	0.509
eGFR (ml/min·1.73 m^2^)	−0.017	0.983 (0.962–1.005)	0.129
Relative percentage decrease in antibody	−0.055	0.947 (0.923–0.971)	**<0.001**
24-h urine protein (g/24 h)	0.314	1.369 (1.120–1.673)	**0.002**
Relative percentage decrease in urine protein	−0.003	0.997 (0.984–1.010)	0.602
Relative percentage increase in albumin	−0.037	0.964 (0.930–1.000)	**0.048**

Bold values indicate statistical significance (*p* < 0.05).

The independently selected factors were re-included in multivariate logistic regression analysis (Table 2), yielding the following predictive model:


$$\begin{array}{l} \text{logit}(p)=2.317–0.057\times (\begin{array}{l} 1-\text{month antibody}\;\\ \text{decrease rate} \end{array})+0.318\\ \times (1-\text{month}\;24-h\;\text{urinary protein excretion})-0.034\\ \times (1-\text{month albumin increase rate}) \end{array}$$

**Table 2:** Multivariate logistic regression analysis of non-remission at 12 months in MN patients: re-inclusion of independent factors based on 1-month treatment outcome screening.

Variable	*β*	Odds ratio (95% CI)	*p*-value
Relative percentage decrease in antibody	−0.057	0.944 (0.922–0.967)	**<0.001**
24-h urine protein (g/24 h)	0.318	1.374 (1.158–1.630)	**<0.001**
Relative percentage increase in albumin	−0.034	0.967 (0.937–0.998)	**0.035**

Bold values indicate statistical significance (*p* < 0.05).

### 3.6 Independent influencing factors after 6 months of treatment

**Figure 3:**
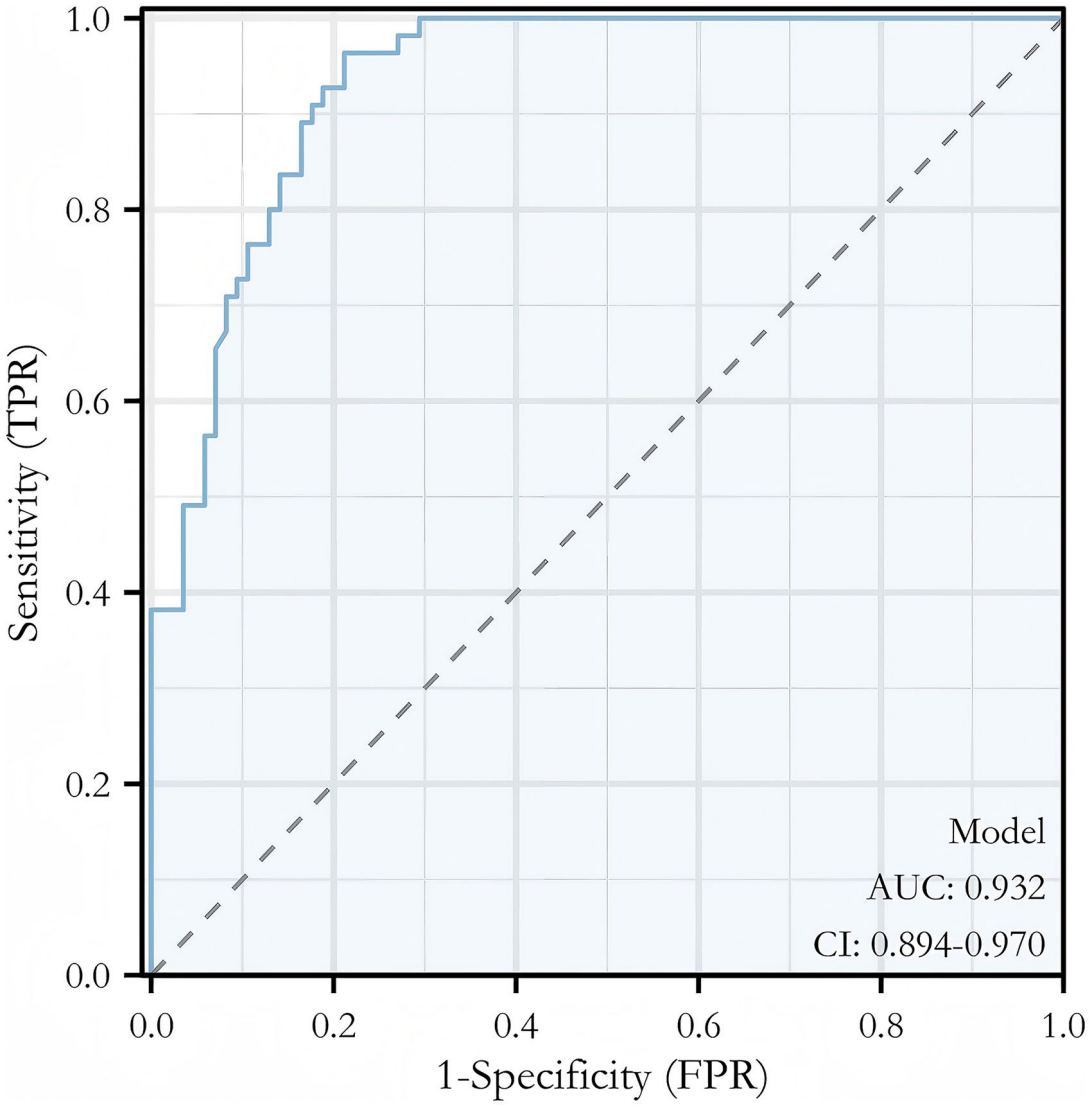
ROC curve of the multivariate logistic regression model with 6-month post-treatment independent factors for predicting 12-month non-remission in MN patients.

After 6 months of treatment, antibodies, 24-h urine protein quantification, albumin, and eGFR were included in multivariate logistic regression analysis. It was found that antibodies not turning negative at 6 months (OR = 8.256, *p* < 0.001), 24-h urine protein quantification (OR = 1.625, *p* < 0.001), and albumin level (OR = 0.868, *p* = 0.043) were independent predictive factors for non-remission after 12 months (Table 3). The AUC of the logistic model constructed was 0.932 (95% CI: 0.894–0.970) (Figure 3).

**Table 3:** Multivariate logistic regression analysis of non-remission at 12 months in MN patients based on 6-month post-treatment data.

Variable	Univariate analysis		Multivariate analysis	
	OR (95% CI)	*p*-value	OR (95% CI)	*p*-value
PLA2R antibody				
Negative	Reference		Reference	
Positive	20.562 (8.595–49.192)	**<0.001**	8.256 (2.793–24.407)	**<0.001**
24-h urine protein (g/24 h)	2.113 (1.638–2.726)	**<0.001**	1.625 (1.223–2.159)	**<0.001**
Albumin level (g/L)	0.740 (0.667–0.820)	**<0.001**	0.876 (0.771–0.996)	**0.043**
eGFR	0.984 (0.972–0.996)	**0.011**	0.987 (0.967–1.008)	0.235

Bold values indicate statistical significance (*p* < 0.05).

### 3.7 Construction and internal validation of the nomogram prediction model

**Figure 4:**
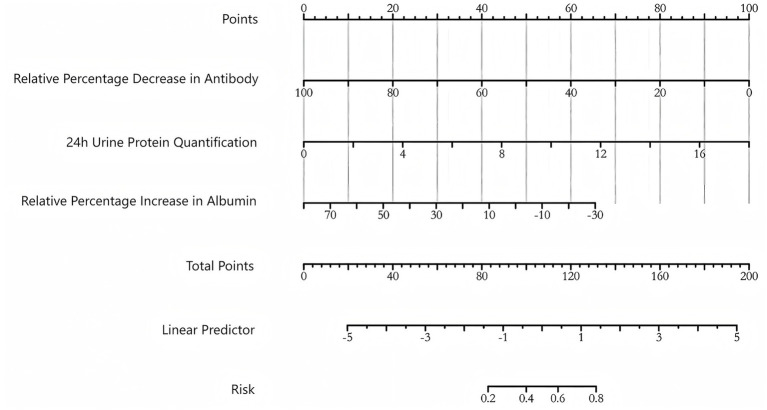
Nomogram prediction model for 12-month non-remission after treatment in MN patients based on independent factors.

A nomogram prediction model for identifying MN patients who do not achieve remission 12 months after treatment was established, incorporating three independent influencing factors: relative percentage decrease in antibodies, 24-h urine protein quantification at 1 month of treatment, and relative percentage increase in albumin (Figure 4). Using the scale provided in the model, individual scores corresponding to each of the three independent influencing factors can be obtained. The total score is calculated by summing the scores of each factor, and the predicted probability corresponding to the total score represents the likelihood that a patient will not achieve remission 12 months later.

**Figure 5:**
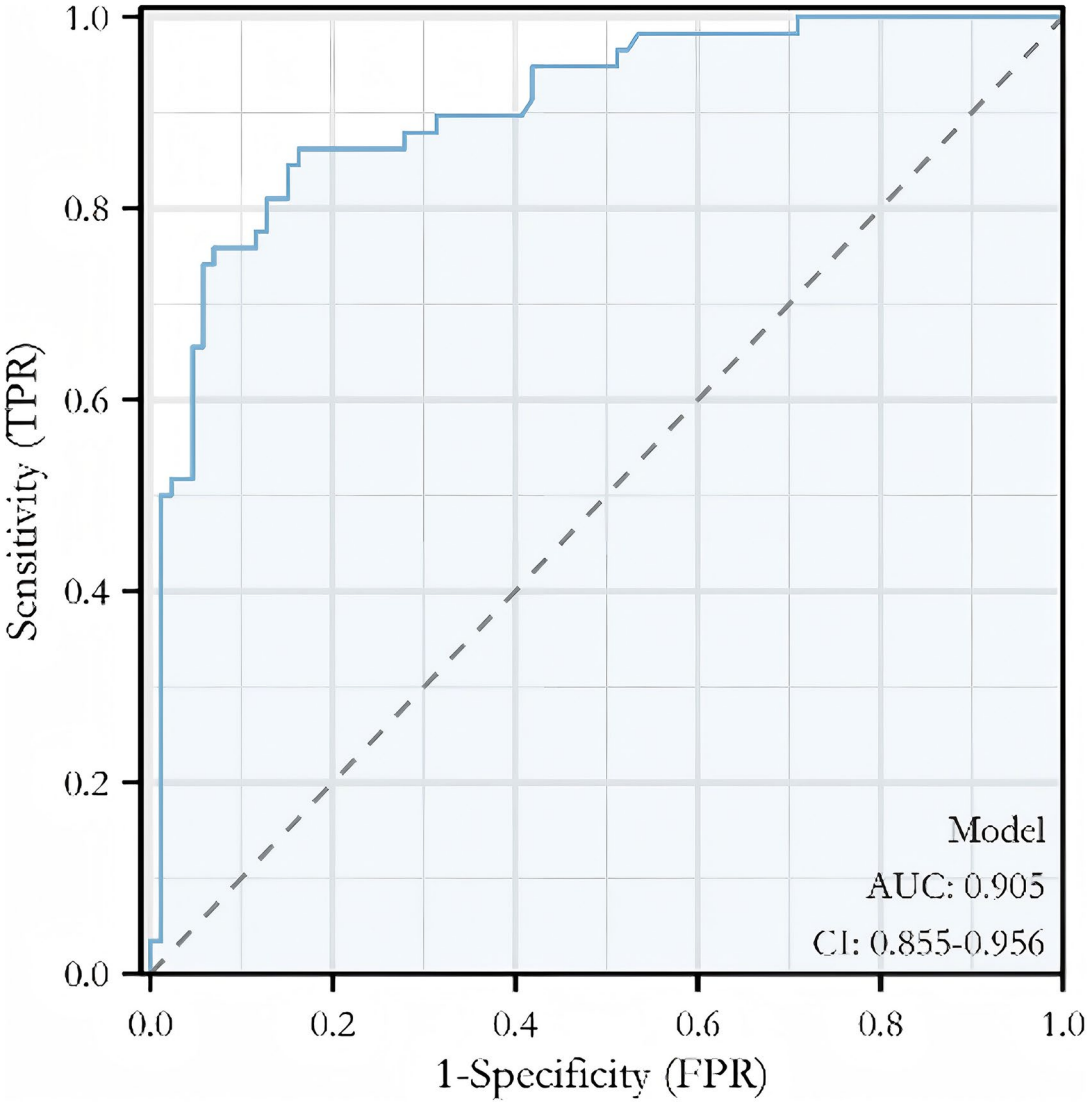
ROC curve of the multivariate logistic regression model at 1-month post-treatment independent factors for predicting 12-month non-remission in MN patients.

The model constructed at 1 month had an AUC of 0.905 (95% CI: 0.855–0.956), indicating good discrimination of the nomogram predictive model (Figure 5). Using the Bootstrap method to repeatedly sample the modeling population data 1,000 times yielded a concordance index (C-index) of 0.906 (95% CI: 0.904, 0.907); 10-fold cross-validation for 1,000 times gave a C-index of 0.898 (95% CI: 0.893, 0.904). Compared to the model at 6 months (AUC: 0.932), there was no significant difference in the model at 1 month (AUC: 0.905). DeLong’s test for paired ROC curves showed no statistically significant difference between the AUC of the 1-month model (0.905, 95% CI: 0.855–0.956) and that of the 6-month model (0.932, 95% CI: 0.894–0.970) (*Z* = 1.04, *p* = 0.2984).

**Figure 6:**
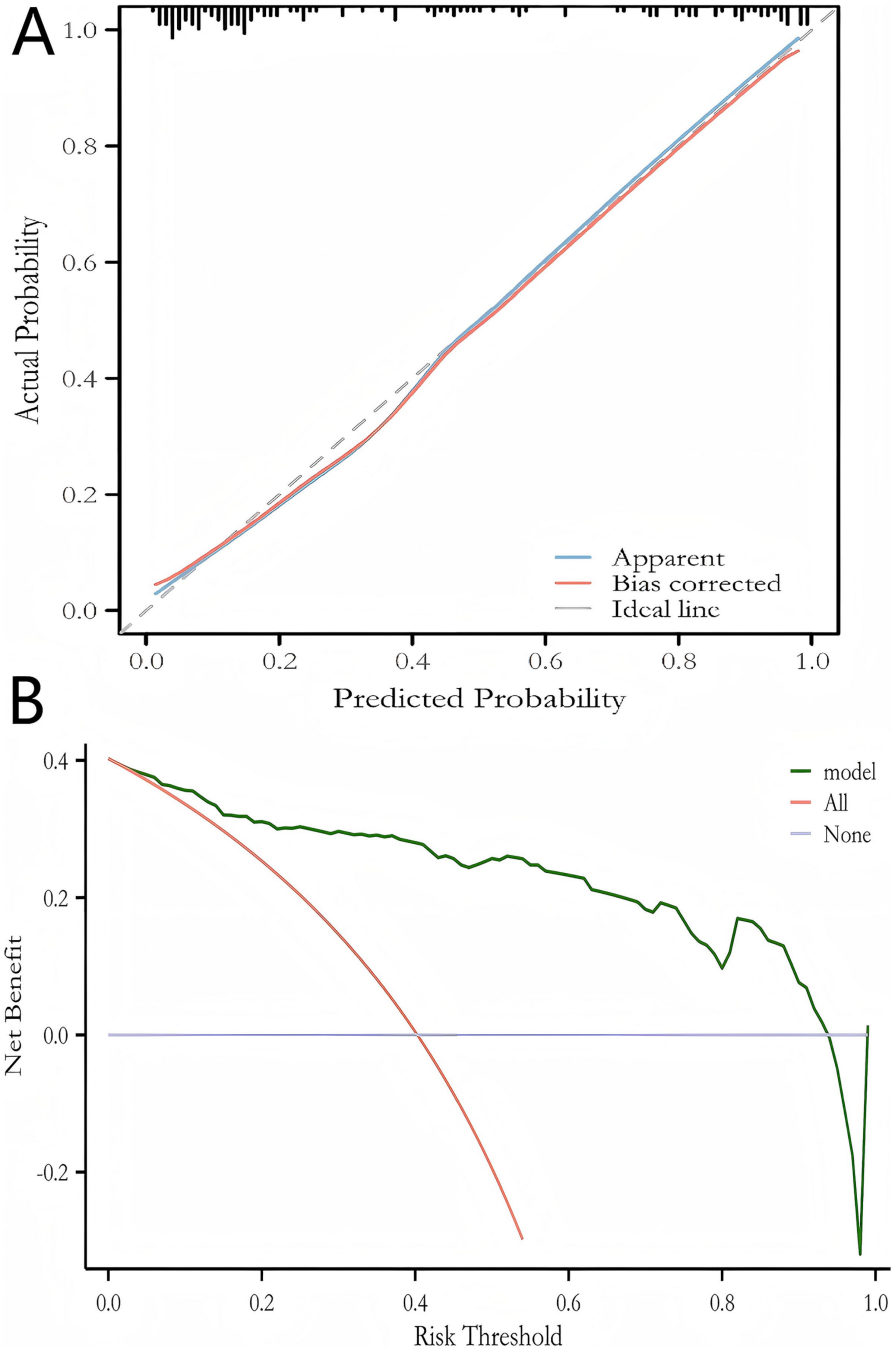
Calibration and clinical efficacy of the nomogram model: Hosmer–Lemeshow test and decision curve analysis. **(A)** Calibration plot of the nomogram for predicting non-remission in MN patients. **(B)** Decision curve analysis of the nomogram for predicting non-remission in MN patients.

The Hosmer–Lemeshow goodness-of-fit test method is used to evaluate the calibration performance of the model. The results show that the Hosmer–Lemeshow test statistic of the model is *χ*^2^ = 7.924, with the corresponding *p*-value being 0.505. This data indicates that the difference between the predicted probabilities and the actual observed results does not reach a statistically significant level (*p* = 0.505 > 0.05), suggesting that the predictive model performs excellently in terms of consistency between probability prediction and actual occurrence, and possesses good calibration performance (Figure 6A). The evaluation of the model using DCA curves indicates that the model constructed in this study has good clinical net benefit (Figure 6B).

## 4 Discussion

This study confirms that 24-h urine protein quantification after 1 month of treatment (OR = 1.374, *p* < 0.001), the relative percentage decrease in anti-PLA2R antibodies (OR = 0.944, *p* < 0.001), and the relative percentage increase in albumin (OR = 0.967, *p* = 0.035) are independent predictors of non-remission at 12 months. Early immune response appears to be a strong predictor of clinical remission. Stefan et al. found that the conversion of anti-PLA2R antibodies to negative within 3 months is an independent predictor of remission, increasing the remission rate by 60% (9). The Ruggenenti team confirmed that a lower baseline anti-PLA2R antibody titer and complete antibody depletion 6 months after RTX treatment strongly predict remission (10), which is consistent with the results of this study. Failure to convert antibodies to negative at 6 months indicates a lower remission rate at 12 months (OR: 8.256, *p* < 0.01), and it was also found that a 50% decrease in antibody titer from baseline is significantly associated with a delayed proteinuria remission (median time difference 3–6 months), suggesting the potential of anti-PLA2R antibody titer as an early predictive factor. The 2021 KDIGO guidelines stratify MN patients into four risk categories (low, moderate, high, and very high) based on a comprehensive assessment of eGFR, serum albumin, urinary protein excretion, anti-PLA2R antibody levels, and the presence of nephrotic syndrome complications. Furthermore, the guidelines define partial remission as a ≥ 50% decrease in proteinuria, underscoring the prognostic value of dynamic changes in proteinuria during treatment (7). Recent findings by Barbour et al. suggest that the dynamic changes in anti-PLA2R antibody levels and albumin levels at 3 months of treatment are the optimal indicators for predicting 12-month outcomes in membranous nephropathy patients, further indicating that dynamic indicators are better than baseline static parameters (such as baseline antibody titer) at reflecting disease activity and treatment response in real time (11). This study advances the monitoring time to 1 month and constructs a model (AUC: 0.905) compared to a model based on static indicators at 6 months (AUC: 0.932), showing no significant difference, suggesting that monitoring time can be advanced to 1 month to identify poor responders earlier and avoid unnecessary prolongation of evaluation time.

By integrating multiple influencing factors, the Nomogram model provides clinicians with an intuitive quantitative prediction tool that can help personalize medical decisions. A variety of nomogram models have been constructed for different aspects of MN, including predicting disease progression, treatment response, likelihood of remission, risk of recurrence, etc. Wen et al. investigated the relationship between serum complement cleavage factor Bb and PMN progression, and established a nomogram model including age, anti-PLA2R antibody, and proteinuria indexes to predict the 3–5 year renal survival rate (C-index 0.77) (12). Wang et al. used a machine learning algorithm to construct a nomogram model, which included the disease course ≥ 6 months, total urine protein (UTP), D-dimer, and anti-PLA2R antibody to predict the prognosis and treatment response of IMN patients (AUROC 0.869) (13). Zhou et al. constructed a nomogram model (AUC 0.721) that included urine protein level and PLA2R-CTLD1-IgG4 level to predict treatment response (14). Hu et al. combined genetic risk scores and anti-PLA2R antibody levels to optimize the prediction model of kidney disease progression of PLA2R-related MN (15). At present, nomogram models on MN mostly rely on baseline static indicators (such as age, eGFR, baseline antibody levels), which cannot reflect the relationship between dynamic changes of indicators and disease activity. Compared with genetic markers (HLA-DQA1) or epitope typing (e.g., CTLD1-IgG4), it may not be suitable for general promotion due to the economic level of each region. This model innovatively integrates the relative percentage of antibody decline, urine protein quantification and albumin recovery relative percentage to construct a nomogram prediction model, which has the advantages of low cost and easy access, and is highly consistent with the concept of focusing on the trend of anti-PLA2R antibody levels rather than the threshold proposed in the 2023 KDOQI commentary on the KDIGO guidelines.

In recent years, RTX has been gradually applied in the treatment of PMN. Compared to traditional treatment regimens, the safety and efficacy of RTX in treating PMN have been confirmed, allowing 60–80% of patients to achieve remission (16–18). However, some patients still fail to achieve remission after RTX treatment. Urinary loss leading to reduced bioavailability, the production of anti-RTX antibodies, and chronic glomerular injury may limit the effectiveness of RTX in treating membranous nephropathy (19).

Reduced bioavailability: Studies indicate that patients with severe nephrotic syndrome exhibit insufficient RTX blood concentrations 3 months after RTX infusion, and the risk increases by 8.66 times for those with baseline serum albumin below 22.5 g/L, as these patients are less likely to achieve clinical and immunological remission (20). Some researchers have found that higher baseline anti-PLA2R antibody levels (>150RU/ml) require larger RTX doses to achieve antibody conversion compared to the low-titer group. Elevated levels of urinary protein (≥3.5 g/24 h) have also been confirmed to be associated with increased anti-PLA2R antibody titers.

Production of anti-RTX antibodies: A study found that 23% of patients tested positive for these antibodies. Patients with antibodies showed faster B-cell reconstitution, higher proteinuria, higher recurrence rates, and a greater need for a second course of RTX treatment. 80% of such antibodies can neutralize RTX activity, and some may also cross-react with new anti-CD20 antibodies. For patients who develop antibodies, humanized anti-CD20 antibodies may be a good alternative therapy (21). Additionally, another study found that risk factors for anti-RTX antibodies include high body weight or BMI > 25 kg/m^2^, high anti-PLA2R antibody titer, high PLA2R1 epitope spreading rate, high IL-17A levels, and low 25-hydroxyvitamin D levels (22). The lack of 25-hydroxyvitamin D is common in nephrotic syndrome, even in the absence of kidney failure. In a randomized controlled trial, after a median follow-up of 5.3 years, vitamin D supplementation significantly reduced the incidence of autoimmune diseases. However, this protective effect gradually disappeared after stopping vitamin D supplementation, and was no longer significant after 2 years of trial follow-up, confirming that vitamin D is involved in immune system regulation (23).

Chronic glomerular injury: The presence of fibrotic glomerular injury may be a cause of significant proteinuria. This will further exacerbate the decline in serum RTX levels. For refractory MN patients resistant to multiple immunosuppressive therapies, it may be difficult to distinguish whether their immunosuppressive resistance is primary or secondary to chronic, irreversible glomerular injury (19).

Therefore, for patients with high baseline anti-PLA2R antibody levels and concurrent severe nephrotic syndrome, a higher dose of monoclonal antibody can be administered initially. Existing studies have confirmed that patients who do not respond to the first course of RTX treatment and do not develop anti-RTX antibodies may respond to repeated RTX treatment (24). For patients who develop anti-RTX antibodies, we should regularly monitor serum RTX levels, anti-drug antibody levels, and 25-hydroxyvitamin D levels, and supplement vitamin D appropriately. Particularly for patients with high body weight or BMI > 25 kg/m^2^, weight reduction may be recommended. If necessary, humanized anti-CD20 monoclonal antibodies can be switched. For patients with chronic glomerular injury, repeated renal biopsies and monitoring of immune activity (anti-PLA2R) may help differentiate patients with immune active diseases, and patients in the active phase may benefit from additional immunotherapy (25). For patients with extensive chronic histological lesions, more education should be provided, and supportive treatment and lifestyle adjustments should be optimized to delay the progression to end-stage renal disease.

It is important to clarify that our study did not use 1-month antibody negativity as a criterion for predicting non-remission. Instead, we employed the percentage decline in anti-PLA2R antibody titers as a continuous variable in the multivariate model. Persistent PLA2R positivity at 1 month should not be interpreted as treatment failure, as longer follow-up is required to adequately assess the immunological response following rituximab therapy. Therefore, while the 1-month model may be useful for early risk stratification, decisions to discontinue or switch therapy should not be based solely on 1-month serological status.

In addition to the above pharmacological strategies for patients identified as likely non-responders at an early stage, alternative and adjunctive approaches are also being actively explored. From an integrative medicine perspective, traditional Chinese medicine (TCM) has shown potential as a promising therapy for patients with membranous nephropathy. Zeng et al. demonstrated that Jianpi Qushi Heluo Formula (JQHF) not only improves renal function but also restores immune homeostasis in MN patients by modulating CD4 + T cell subsets, particularly shifting the inflammatory profiles of Th1/Th17 cells toward Th2/Treg (26). However, it should be noted that these findings are preliminary, and larger-scale controlled trials are needed to establish JQHF as a viable therapeutic option. Furthermore, emerging research has highlighted the gut microbiota-MN axis, suggesting that dysbiosis of the gut microbiome may influence immune regulation and contribute to the pathogenesis and progression of MN. Zhao et al. reviewed recent advances in the gut–kidney axis in MN and explored new therapeutic interventions targeting gut microbiota modulation, including probiotics, prebiotics, fecal microbiota transplantation, and natural product therapeutic strategies (27). Nevertheless, research in this area is still in its early stages, and whether microbiota-targeted interventions can improve outcomes in MN patients, particularly those who are refractory to standard immunotherapy, requires further investigation. It is important to clarify that these alternative perspectives are presented as contextual background and future research directions, not as direct evidence supporting the rituximab-based nomogram developed in this study. They do not replace current evidence-based immunosuppressive therapies but may offer complementary avenues for future research and personalized management.

This study used a single-center retrospective design and has certain limitations. On one hand, due to severe missing follow-up data at 3 months after treatment (approximately 85% of patients had incomplete data at this time point), clinical indicators during this period were not included in the study. Complete case analysis was used for the 1-month and 6-month time points, where missing data were minimal (<5%). We acknowledge that the exclusion of the 3-month time point may have resulted in the loss of potentially informative early response data. However, because missingness was primarily due to inconsistent follow-up scheduling rather than patient clinical characteristics, the likelihood of significant bias is low. Furthermore, baseline characteristics did not differ significantly between patients with and without complete data. Nevertheless, the absence of 3-month data may affect the completeness of the longitudinal response trajectory and should be addressed in future prospective studies with standardized follow-up protocols. On the other hand, the study only included cases receiving RTX treatment, and this selection method may lead to selection bias, limiting the generalizability of the study results. Although all patients received standardized supportive care as described in the Methods, we cannot completely exclude the possibility that differences in the intensity of RAASi titration or diuretic use may have influenced proteinuria and albumin levels, which are key inputs to our prediction model. However, supportive therapy was administered according to a uniform institutional protocol, minimizing potential bias. Additionally, this study did not undergo external validation. Furthermore, baseline pathological data, including glomerulosclerosis and interstitial fibrosis/tubular atrophy (IFTA), were not included in our analysis. According to the 2021 KDIGO guidelines, kidney biopsy is not mandatory for diagnosing PLA2R-related MN in patients with positive serum anti-PLA2R antibodies and typical clinical features of nephrotic syndrome; therefore, biopsies were not routinely performed in our cohort to avoid unnecessary invasive procedures and reduce patient burden. Consequently, histologic chronicity variables were unavailable. We acknowledge that the absence of these pathological data may limit the generalizability of our model and could introduce unmeasured confounding. However, all included patients had relatively preserved baseline eGFR (mean >60 mL/min per 1.73 m^2^), partially mitigating concerns about advanced chronic histologic changes in this specific cohort. Additionally, emerging prognostic factors such as anti-PLA2R antibody epitope spreading were not assessed due to the retrospective design of this study. To establish a more robust and applicable prognosis model for MN in Chinese patients, future multicenter, large-sample prospective studies are needed. Through multicenter collaboration, the sample size can be expanded to cover a broader patient population and reduce selection bias. Future studies should also incorporate standardized pathological assessments and epitope analysis to validate and potentially refine our nomogram model. In summary, after 1 month of treatment, the relative percentage decrease in antibody levels and the relative percentage increase in albumin in 24-h urine protein quantification are independent influencing factors for non-remission after 12 months, and can serve as predictive factors for the prognosis of MN. Compared to monitoring at the 1st month after treatment, there was no significant disadvantage in predictive effect when monitoring at the 6th month. The nomogram model constructed based on this can effectively predict the risk of non-remission in MN patients after 12 months of treatment. Therefore, during early follow-up of patients, these indicators should be closely monitored, and timely intervention should be provided to improve the remission rate in MN patients.

## 5 Conclusion

This study confirms that the 24-h urinary protein quantification, the percentage decrease in antibodies, and the percentage increase in albumin after 1 month of treatment are independent influencing factors for predicting non-remission in patients with MN after 12 months. The nomogram prediction model established based on this, when only using the monitoring data of the first month, has a predictive performance comparable to that of the sixth month data. It can realize individualized quantitative assessment of the risk of non-remission at 12 months, providing a practical guidance tool for clinical early intervention decisions.

## Data Availability

The original contributions presented in the study are included in the article/Supplementary material, further inquiries can be directed to the corresponding author.

## Supplementary material

SUPPLEMENTARY TABLE 1 Comparison of baseline clinical data between response and non-remission groups before treatment.

SUPPLEMENTARY TABLE 2 Comparison of clinical data between remission and non-remission groups after 1 month of treatment.

SUPPLEMENTARY TABLE 3 Comparison of clinical data between remission and non-remission groups after 6 months of treatment.

SUPPLEMENTARY TABLE 4 Univariate and multivariate logistic regression analysis of baseline pretreatment factors associated with non-remission at 12 months in MN.
