# Supplementary material for: Predicting membranous nephropathy remission: a nomogram based on early dynamic biomarkers
Source: Front Med (Lausanne). 2026 Jul 20;13:1783016. doi: 10.3389/fmed.2026.1783016 (PMC13430465; doi:10.3389/fmed.2026.1783016)
Supplement: SUPPLEMENTARY TABLE 1 — Comparison of baseline clinical data between response and non-remission groups before treatment. [file Table_1.DOCX]

| Variable | Remission Group n=86 | Non - remission Group n=58 | P - value |
| --- | --- | --- | --- |
| Gender |  |  | 0.578 |
| Female | 29 (20.1%) | 17 (11.8%) |  |
| Male | 57 (39.6%) | 41 (28.5%) |  |
| Age | 52.372 ± 11.477 | 56.379 ± 11.607 | **0.043** |
| BMI(kg/m²) | 24.84 (23.275, 28.650) | 26.33 (24.137, 29.617) | 0.155 |
| Hypertension |  |  | **0.038** |
| No | 30 (20.8%) | 11 (7.6%) |  |
| Yes | 56 (38.9%) | 47 (32.6%) |  |
| Diabetes |  |  | 0.752 |
| No | 81 (56.2%) | 53 (36.8%) |  |
| Yes | 5 (3.5%) | 5 (3.5%) |  |
| Antibody Titer Group |  |  | **0.023** |
| <150 RU/ml | 67 (46.5%) | 35 (24.3%) |  |
| >150 RU/ml | 19 (13.2%) | 23 (16.0%) |  |
| 24h Urine Protein (g/24h) | 5.500 (3.8625, 7.9850) | 5.805 (4.0200, 8.7825) | 0.381 |
| Albumin (g/L) | 25.82 (22.217, 28.942) | 24.89 (19.392, 28.470) | 0.153 |
| Triglyceride (mmol/L) | 2.115 (1.6825, 3) | 2.415 (1.79, 3.85) | 0.323 |
| Total Cholesterol (mmol/L) | 6.585 (5.3825, 8.6275) | 6.875 (5.485, 9.945) | 0.183 |
| Uric Acid (μmol/L) | 382.630 ± 90.476 | 401.340 ± 114.070 | 0.299 |
| Urea (mmol/L) | 4.995 (4.0925, 6.3125) | 5.170 (4.415, 7.055) | 0.298 |
| D-dimer (ng/ml) | 241 (142, 407) | 240 (134, 414) | 0.932 |
| Serum Creatinine (μmol/L) | 69.15 (59.2, 81.85) | 73.20 (61.70, 90.20) | 0.106 |
| eGFR (ml/min·1.73㎡) | 106.43 (96.095, 120.8) | 101.00 (77.11, 113.8) | 0.061 |
| Platelet Count (×10⁹/L) | 261.55 ± 66.860 | 251.66 ± 71.364 | 0.398 |
| Neutrophil Count (×10⁹/L) | 4.03 (3.14, 5.15) | 3.76 (3.0775, 5.145) | 0.642 |
| Lymphocyte Count (×10⁹/L) | 1.865 (1.55, 2.4875) | 1.790 (1.465, 2.3375) | 0.408 |
| SII | 534.96 (396.62, 797.08) | 501.13 (387.04, 741.55) | 0.559 |
| NLR | 2.1158 (1.5402, 3.3376) | 2.1547 (1.5466, 3.1657) | 0.783 |
| PLR | 128.44 (108.35, 180.23) | 120.75 (103.44, 185.57) | 0.644 |
| Hemoglobin (g/L) | 138.77 ± 18.901 | 134.91 ± 21.237 | 0.258 |
