# Supplementary material for: Predicting membranous nephropathy remission: a nomogram based on early dynamic biomarkers
Source: Front Med (Lausanne). 2026 Jul 20;13:1783016. doi: 10.3389/fmed.2026.1783016 (PMC13430465; doi:10.3389/fmed.2026.1783016)
Supplement: SUPPLEMENTARY TABLE 2 — Comparison of clinical data between remission and non-remission groups after 1 month of treatment. [file Table_2.DOCX]

| variables | Remission Group | Non-remission Group | P-value |
| --- | --- | --- | --- |
| PLA2R Antibody |  |  | **<0.001** |
| Negative | 23 (16%) | 3 (2.1%) |  |
| Positive | 63 (43.8%) | 55 (38.2%) |  |
| Antibody Level (RU/ml) | 10.245 (2.5, 22.89) | 51.020 (26.947, 109.59) | **<0.001** |
| **24-hour Urine Protein** (g/24h) | 3.365 (1.98, 4.155) | 6.555 (4.2, 8.6325) | **<0.001** |
| Albumin Level (g/L) | 31.28 (26.502, 34.48) | 26.65 (20.57, 31.197) | **<0.001** |
| Relative Percentage Decrease in Antibody (%) | 85.735 (76.314, 92.383) | 47.264 (33.105, 67.715) | **<0.001** |
| Relative Percentage Decrease in Urine Protein (%) | 42.4620 (15.037, 57.23) | 4.5167 (-33.949, 26.432) | **<0.001** |
| Relative Percentage Increase in Albumin (%) | 17.059 (8.7277, 31.406) | 6.472 (-0.27449, 14.964) | **<0.001** |
| Absolute Change in Antibody (RU/ml) | 56.025 (33.602, 114.84) | 39.150 (15.852, 121.86) | 0.075 |
| Change in Albumin (g/L) | 4.7910 ± 3.8242 | 1.8943 ± 3.3938 | **<0.001** |
| Change in 24h Urine Protein (g/24h) | 1.955 (0.59, 4.09) | 0.200 (-2.12, 1.58) | **<0.001** |
