# Supplementary material for: Predicting membranous nephropathy remission: a nomogram based on early dynamic biomarkers
Source: Front Med (Lausanne). 2026 Jul 20;13:1783016. doi: 10.3389/fmed.2026.1783016 (PMC13430465; doi:10.3389/fmed.2026.1783016)
Supplement: SUPPLEMENTARY TABLE 3 — Comparison of clinical data between remission and non-remission groups after 6 months of treatment. [file Table_3.DOCX]

| Variable | Remission Group | Non-remission Group | P-value |
| --- | --- | --- | --- |
| PLA2R Antibody |  |  | <0.001 |
| Negative | 51 (47.2%) | 9 (8.3%) |  |
| Positive | 12 (11.1%) | 36 (33.3%) |  |
| **24-hour Urine Protein** (g/24h) | 1.23 (0.665, 2.0625) | 4.83 (3.075, 8.115) | <0.001 |
| Serum Albumin (g/L) | 37.122 ± 5.0962 | 30.063 ± 5.8372 | <0.001 |
| eGFR (ml/min·1.73㎡) | 113.18 (94.48, 129.66) | 108.82 (83.87, 114.99) | 0.028 |
| Relative Percentage Decrease in Antibody (%) | 95.132 (91.737, 97.162) | 84.951 (61.436, 93.709) | <0.001 |
| Relative Percentage Decrease in Urine Protein (%) | 78.739 (62.237, 87.489) | 15.455 (-17.938, 44.167) | <0.001 |
| Relative Percentage Increase in Albumin (%) | 41.575 (27.865, 59.964) | 14.505 (5.6982, 27.279) | <0.001 |
| Absolute Change in Antibody (RU/ml) | 61.170 (35.32, 129.74) | 60.845 (31.51, 170.45) | 0.703 |
| Change in Albumin (g/L) | 41.575 (27.865, 59.964) | 14.505 (5.6982, 27.279) | <0.001 |
| Change in 24h Urine Protein (g/24h) | 4.195 (1.8675, 5.94) | 0.630 (-0.815, 2.215) | <0.001 |
