# Supplementary material for: Predicting membranous nephropathy remission: a nomogram based on early dynamic biomarkers
Source: Front Med (Lausanne). 2026 Jul 20;13:1783016. doi: 10.3389/fmed.2026.1783016 (PMC13430465; doi:10.3389/fmed.2026.1783016)
Supplement: SUPPLEMENTARY TABLE 4 — Univariate and multivariate logistic regression analysis of baseline pretreatment factors associated with non-remission at 12 months in MN. [file Table_4.DOCX]

| Variable | Univariate analysis | |  | Multivariate analysis | |
| --- | --- | --- | --- | --- | --- |
|  | OR (95% CI) | P-value |  | OR (95% CI) | P-value |
| **24-hour Urine Protein** (g/24h) | 1.036(0.960 – 1.117) | 0.361 |  |  |  |
| Albumin (g/L) | 0.953 (0.893 – 1.017) | 0.148 |  |  |  |
| PLA2R Antibody |  |  |  |  |  |
| <150 RU/ml | Reference |  |  | Reference |  |
| >150 RU/ml | 2.317 (1.114 – 4.820) | 0.024 |  | 2.635 (1.162 – 5.978) | 0.020 |
| Gender |  |  |  |  |  |
| Female | Reference |  |  |  |  |
| Male | 1.227(0.597 – 2.523) | 0.578 |  |  |  |
| Age | 1.031(1.001 – 1.062) | 0.045 |  | 1.016 (0.981 – 1.052) | 0.370 |
| BMI(kg/m²) | 1.036 (0.960 – 1.119) | 0.359 |  |  |  |
| Diabetes |  |  |  |  |  |
| No | Reference |  |  |  |  |
| Yes | 1.528 (0.422 – 5.536) | 0.518 |  |  |  |
| eGFR (ml/min·1.73㎡) | 0.983 (0.970 – 0.996) | 0.013 |  | 0.981 (0.965 – 0.998) | 0.025 |
| Triglyceride (mmol/L) | 0.981 (0.913 – 1.054) | 0.603 |  |  |  |
| Total Cholesterol  (mmol/L) | 1.127 (1.034 – 1.228) | 0.007 |  | 1.135 (1.034 – 1.246) | 0.008 |
| Uric Acid  (μmol/L) | 1.002 (0.999 – 1.005) | 0.276 |  |  |  |
| Urea(mmol/L) | 1.121(0.970 – 1.297) | 0.122 |  |  |  |
| D-dimer(ng/ml) | 1.000 (1.000 – 1.001) | 0.233 |  |  |  |
| Hypertension |  |  |  |  |  |
| No | Reference |  |  | Reference |  |
| Yes | 2.289(1.037 – 5.055) | 0.040 |  | 2.606(1.073 – 6.328) | 0.034 |
